# Whole Genome Sequencing Applied to Pathogen Source Tracking in Food Industry: Key Considerations for Robust Bioinformatics Data Analysis and Reliable Results Interpretation

**DOI:** 10.3390/genes12020275

**Published:** 2021-02-15

**Authors:** Caroline Barretto, Cristian Rincón, Anne-Catherine Portmann, Catherine Ngom-Bru

**Affiliations:** Institute of Food Safety and Analytical Sciences, Nestlé Research, 1000 Lausanne 26, Switzerland; cristian.rincon@rd.nestle.com (C.R.); annecatherine.portmann@gmail.com (A.-C.P.); catherine.ngombru@rdls.nestle.com (C.N.-B.)

**Keywords:** whole genome sequencing, food industry, bioinformatics, workflow, data analysis, metadata, food safety, data quality

## Abstract

Whole genome sequencing (WGS) has arisen as a powerful tool to perform pathogen source tracking in the food industry thanks to several developments in recent years. However, the cost associated to this technology and the degree of expertise required to accurately process and understand the data has limited its adoption at a wider scale. Additionally, the time needed to obtain actionable information is often seen as an impairment for the application and use of the information generated via WGS. Ongoing work towards standardization of wet lab including sequencing protocols, following guidelines from the regulatory authorities and international standardization efforts make the technology more and more accessible. However, data analysis and results interpretation guidelines are still subject to initiatives coming from distinct groups and institutions. There are multiple bioinformatics software and pipelines developed to handle such information. Nevertheless, little consensus exists on a standard way to process the data and interpret the results. Here, we want to present the constraints we face in an industrial setting and the steps we consider necessary to obtain high quality data, reproducible results and a robust interpretation of the obtained information. All of this, in a time frame allowing for data-driven actions supporting factories and their needs.

## 1. Introduction

Advances in DNA sequencing technologies have transformed the capacity to investigate the dynamics of foodborne pathogens inhabiting diverse environments. Food industry [1,2] and food safety agencies [3,4,5] have benefited from these improvements for pathogen source tracking. Whole genome sequencing (WGS) has arisen as a tool with a scope that goes beyond academic research proving to be an asset in ensuring food safety. The technologies have gone through considerable improvements in recent years making them both faster and cheaper, allowing thus for relatively wide adoption and use for food safety and public health on a routine basis [6]. Multiple governmental entities and regulatory authorities have established networks coordinating the efforts of nationwide laboratories to use WGS for foodborne pathogen source tracking [3,7,8] and real-time outbreak detection and investigation [9,10].

Nowadays, WGS is widely considered as the approach offering the highest resolution and precision for routine real-time surveillance concerning foodborne pathogens and has been adopted by multiple regulatory agencies worldwide [6,11,12,13]. This has been accompanied by the development of several computational software and methods allowing the analysis of these data. Nevertheless, a wider adoption of the technology, particularly in food industry, has been lessened by several factors [14,15]. First, the computational infrastructure required to process (and store) the data. Moreover, little consensus exists concerning the use of the multitude of software available to process sequencing data, and although general guidelines are being consolidated, to date, multiple institutions use different internally validated workflows [16,17]. Despite advancements in recent years, the speed in which useful information can be provided to the factories has also limited the adoption of WGS for foodborne pathogen surveillance in industry. Timewise, collecting the samples, sending them for identification and sequencing is a major limiting factor. Additionally, the defined workflow for genome analysis should produce interpretable results within a time frame that does not limit their operational applicability. Finally, the added value of WGS over traditionally used typing methods relies on an accurate interpretation of the results. An analysis conducted using WGS is a powerful tool for determining the relatedness of bacterial isolates in pathogen source tracking. However, by itself, it can only indicate that isolates recently arose from the same source. Linking the information obtained via WGS with the likely origin of the foodborne contaminant requires contextualization of the results using information about the sample (metadata), thus requiring appropriate expertise. Detection of a pathogen or its relatedness to others, might not be enough to identify the root cause of the contamination. WGS has the power to provide information exploitable by quality managers that can be used to develop data-driven strategies for food safety management. 

Over the years, guidelines for the standardization of wet lab protocols have been issued, however, for data processing, there is still a large gap to close regarding standardization to ensure the analysis is complete and correct, for ultimately making an accurate recommendation. Deep understanding of the results is key in order to support factories in their root cause analysis investigations. Software will always provide a result, however without critical review and appropriate verifications, these results can be erroneous and lead to incorrect interpretations. All in all, the integration of microbiology, genomics and bioinformatics knowledge is essential to ensure the quality of the data, validate the analytical results and provide a reliable interpretation of the obtained information, integrating metadata. Several bioinformatics approaches have been tested and validated [18,19,20] and here, more than introducing a workflow, we present what we consider important as key considerations for robust bioinformatics data analysis and reliable results interpretation in the context of whole genome sequencing applied to pathogen source tracking in food industry.

## 2. Materials and Methods

### Workflow

The data analysis workflow was split into stages and, for each of them, quality control metrics were defined to ensure high quality of the obtained data. Based on selection criteria, several open-source software were identified for each step, tested and benchmarked. 

The considered criteria for software selection were to be open-source and well documented, Linux-based, actively maintained, locally installable (i.e., not running in external or cloud servers), compliant with internally defined IT security regulations, adapted to be launched on large datasets and fast enough to allow the whole workflow to be run within 24 h on a dedicated server. Importantly, these tools can be used for a large range of pathogens. Here we present our experiences notably with *Listeria monocytogenes* and *Salmonella enterica*.

For each stage, the best performing software (for our specific case), fulfilling the above criteria, was streamlined into a workflow. This selection was based on accuracy and reproducibility of the results during the benchmarking. For each stage, several metrics from the output data were considered as quality checks. When an isolate failed one of these quality checks, it was tagged as of low quality to be further examined. As bioinformatics software are rapidly changing and developing, the intention of this work was not to promote a certain software or benchmark its performance but rather highlight the criteria to be considered to ensure high quality data for downstream analyses. Table 1 summarizes the stages and metrics considered. 

We also added, among others, data integrity verification with the md5sum software when data are transferred between servers and cross-referencing isolate identification with metadata. Importantly, the criteria mentioned here are fit-for-purpose for Illumina data. Long read data (Pacific Biosciences or Oxford Nanopore) will require adjustments. 

## 3. Metadata

Metadata, understood as the collection of information related to each sample and its processing, is key for results interpretation. These data (factory name, internal isolate number, sampling time, material type, country, physical description of the sample, sampling point, isolate taxonomy, DNA extraction kit, sequencing chemistry, sequencing platform, operator, etc.) need to be managed and curated to ensure its quality and utility for results interpretation. Thus, data quality checks and data management practices apply to metadata collection and storage. This kind of information is often handled via a laboratory information management system (LIMS). A LIMS frequently includes sample tracking, stock management and data exchange interfaces. As for any other database, metadata management requires resources, quality monitoring, data backup and recovery and security compliance. The more data is acquired, the more accurate the data interpretation will be. Different stakeholders in the food industry have a key role to collect and manage this metadata from the person who routinely takes the swabs/samples, to the laboratory that does the initial diagnostic and then to the WGS lab. Therefore, controls are needed to operate the flow of data and the data management should be considered as an integral and key component of any WGS analysis workflow.

## 4. Results

**Figure 1:**
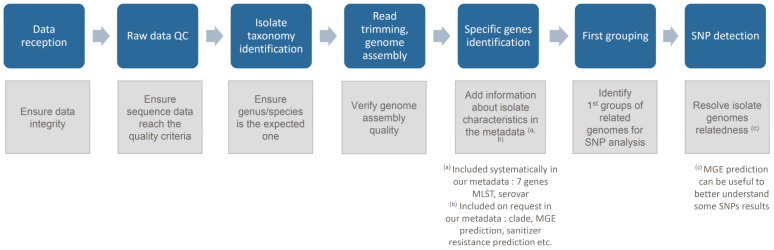
Key components of our pathogen source tracking whole genome sequencing (WGS) workflow: Overview and main goal of each step.

A stepwise approach is recommended to fully control the analysis and can be described in key components (Figure 1). 

The software included in our workflow are listed in Table 2. The rationale for the choice of each software implemented in our workflow was a combination of: (i) the possibility of the software to produce in their output the parameters that are used as quality control (QC) evaluation (mentioned in Table 1); (ii) features mentioned in material and methods, and performance such as accuracy and precision.

Several of these conditions may be less relevant in another setting (e.g., cloud computing is a valid option when there is no sensitive data). Thus, the choice of tools is context specific.

Initial data integrity verification is important since data corruption may occur during transfer from the sequencing server to the analysis server, due to, for example, network interruptions. Once a sequencing run is ready to be analyzed, key quality verifications are necessary to make sure the information of each sample can be used appropriately. 

The quality verification of the fastq files is done to ensure that sequencing reads have the appropriate size and nucleotide quality for each position. At this stage, the estimated genome average coverage and the GC content are also evaluated. Having an appropriate genome average coverage ensures not only accurate Single Nucleotide Polymorphism (SNP)/allele calling, but also high-quality genome assemblies. A GC content not concordant with the expected genus can also be a good indicator of a possible contamination and should be examined. Including a negative control, meaning a sample that is supposed to be DNA free, could be a good practice in the sequencing laboratory. A high number of reads in the negative sample indicates contamination of the lab supplies with foreign DNA and therefore a contamination of the sequenced samples. Adding a negative control in the sequencing run would have no impact on the sequencing costs and should not preempt other isolates sequencing coverage since this sample is supposed to be DNA free. Similarly, a positive control should be included whenever possible since it can help for troubleshooting a failed run. For example, a suboptimal result in terms of reads throughput (including the positive control) points to issues with the library preparation or the sequencing chemistry quality. This can make a difference on the ability to maintain turnaround time of the sequencing and reduce consumables used in troubleshooting.

After assembling the sequencing reads into a genome, a low number of contigs is one of the indications of high quality of the assembly. The overall genome size should enter within the expected size for the predicted genus [30]. Furthermore, for better reproducibility, a software producing an identical assembly for the same input when ran multiple times is advantageous [28]. As a laboratory often handles multiple sample types, it is not exceptional that a sample mix or a contamination occurs when extracting DNA or preparing sequencing libraries. It is thus essential to accurately assign a taxonomy to the sequenced isolate and to have an indication of potential contamination from other samples. 

The additional information that can be obtained from whole genomes such as the serovar prediction for *Salmonella*, the seven genes Multi-Locus Sequence Type (MLST) prediction for a large number of organisms, mobile genetic element detection such as plasmids or phages, is highly valuable for a reliable data interpretation.

Before starting SNP analysis, a key step is to make sure that only closely related genomes are included in the analysis. The variant calling for SNP analyses relies in comparisons to a reference genome and the choice and quality of this reference impacts the obtained information [42]. Comparing genetically distant genomes, implies having portions of the genomes with low read mapping and potentially generating SNP hotspots (several SNPs in close proximity). As both, low mapping and SNP dense regions, are generally excluded from the analyses, SNPs at those positions are excluded too, leading to artificially low differences and to a false interpretation of the results. At this point, having the possibility to quickly compare large datasets in a short time performing a first grouping with, for instance, core genome/whole genome MLST (cg/wgMLST), enables to focus on groups of isolates that are related to then be further separated by SNP analyses. If during the allele call, a locus is not found in a genome, it is zeroed and removed from the analysis. When this occurs for a large number of loci, similarity among the compared strains will be artefactually increased. Hence, it is important to also have a critical eye on the allele call quality and to further analyze the groups of related genomes with a SNP approach.

Even after implementing quality checks, a workflow may need several rounds of analyses to be completed. In some cases, SNPs hotspots are identified and frequently these SNPs are present on mobile genetic elements (MGE), such as phages and plasmids. As these variants are not phylogenetically relevant [6,43], they are often filtered out in the final analyses. An MGE identifier pipeline should be coupled with SNP analyses to verify whether these hotspots are due to the presence of MGEs and not because of low quality of the reference genome assembly or low mapping of reads versus the reference genome. 

Finally, a crucial step for results interpretation is to contextualize the findings using the metadata. Its utility greatly depends on the implementation of a controlled vocabulary to ensure consistency among contributors to the database (e.g., factory, laboratory and sequencing facility). During the process of standardization, it is key to consider the requirements of bioinformaticians (or whomever must interpret the results of genomic analysis), microbiologists and quality managers to define the information to be captured. In recent years, several publications have addressed the minimum information required to process sequencing data [44,45]. Reliability of metadata is essential for its proper use in the interpretation of a WGS analysis. The importance for standardized, high-quality metadata, called for implementation of practices as the development of food safety specific ontologies [46,47] and, more recently, ontologies relevant to bioinformatics analyses in food safety [48]. These procedures aim to make the metadata accessible, exchangeable, and minable as well as to ease the information control and flow. 

## 5. Discussion

Rigorous environmental monitoring in food factories aims at verifying microbiological food safety control measures and detection of a potential pathogen contamination before it reaches the product. WGS has proven to be of high sensitivity and is routinely used by authorities for pathogen source tracking in outbreak events. Similarly, WGS can be used in root cause investigation in case of factory pathogen contamination. Data produced with WGS by itself cannot provide an answer and needs to be interpreted and contextualized accordingly to the biological question asked. Additional discriminatory power of WGS analyses requires also a higher degree of expertise to understand and interpret the results. In order to obtain robust results, high quality data and analysis are required. Establishing several filters and metrics evaluation for raw data, genome assemblies and typing analyses avoid the inclusion of low-quality data that can impact the results and the subsequent interpretation. This not only aligns with the aim to obtain reliable results from the first attempt but also contributes to standardization and automation of such workflows. Capitalizing on hands-on experience to establish metrics that allow for high quality data, can also contribute to optimization of resources use (particularly time) and the obtention of interpretable information. A strong understanding of the bacterial genomics and the bioinformatics analysis is important to correctly interpret the results.

### 5.1. Turnaround Time

A long analysis turnaround-time (TAT) greatly limits the usability of the information generated via WGS. Implementing quality checks at several stages of the analyses not only increases the value of the analysis but also optimizes time use by having a “first time right” analysis. Every additional examination due to *a posteriori* corrections for low quality data increases TAT. Integrating quality checks in our workflow has allowed to reduce the bioinformatics analysis TAT by more than 80% (reaching in average ~1 day) and to provide timely results to factories.

### 5.2. cg/wgMLST versus SNP

It is widely recognized that cg/wgMLST and SNP analyses are highly concordant [49,50,51,52,53] for pathogen source tracking. However, contrary to cg/wgMLST, SNP typing allows for identification of mutations in the non-coding regions of the genome. This allows for the use of almost all the genetic information from a genome thus providing the theoretically highest level of precision available. This additional information may explain the cases in which the cg/wgMLST and SNP approaches were not concordant [50,54]. We have observed many cases in which cgMLST did not provide a clear-cut answer where SNP was able to fully resolve relatedness. 

Combining additional information of the sample (metadata) cgMLST and SNP analyses allows formulation of data-driven actions for controlling the actual source of the contaminant and strategies to avoid the contaminant to ever reach the final product.

### 5.3. Standardization and Accreditation

International efforts are being carried out to harmonize protocols not only in the lab but also in the bioinformatics analysis. In an ISO working group, lab specialists, microbiologists, bioinformaticians and metadata experts are actively working on the international ISO 23418 standard (whole genome sequencing for typing and genomic characterization of foodborne bacteria) offering general guidelines (https://www.iso.org/standard/75509.html (accessed on 16 December 2020)). 

Laboratory proficiency tests have been developed in Europe and in USA to monitor the WGS lab work, and are now running on a regular basis [55]. Proficiency tests for bioinformatics analysis for pathogen source tracking are less frequent, and more complicated to design due to lack of generalized guidelines. However, in recent years some have been developed and used [56,57]. This lack of consensus for data analysis highlights the importance of metrics evaluation and workflow validation [18,19,20] to ensure its quality.

Additionally, efforts towards standardization of the several steps in WGS bioinformatics analyses will greatly contribute to the results reproducibility and portability of the workflows. Open-source command-line software running in a high-performance computing infrastructure is often seen as the best way to perform these analyses. Standardization and data quality assurance, by optimizing resources use, could also enable the use of packaged workflows (e.g., Docker https://github.com/docker/docker-ce (accessed on 18 December 2020)) for smaller infrastructures or where cloud-computing is a possibility if IT security allows.

### 5.4. Internal Genome Database and Metadata Management

Having an established computing infrastructure allows also for maintaining an internal database of sequenced isolates. Although this requires data management, it offers multiple benefits. Firstly, it permits cross-referencing genomes from current and previous case studies to identify potential links between and within factories, raw materials or any information stored in the metadata. An example of this could be the case of a contaminant found in two, otherwise unrelated, facilities where the link is a shared raw material. These kinds of scenarios are by themselves a good justification for the use of an internal metadata database linked with WGS analysis. Furthermore, the sequencing data may be used for genome mining, definition of in-house allelic profiles (cg/wgMLST), antibiotic resistance surveys, mutation rates studies, among others.

Finally, having full genome information allows for other kinds of research that could ultimately benefit the food industry, for example, studying the effects of cleaning agents on microbial resistance to biocides [58,59,60,61]. Additionally, complete genome sequences can be used to predict antimicrobial resistance genes or virulence genes presence [62,63]. 

### 5.5. Analysis Reproducibility and Repeatability

As mentioned before, the fast advancements on sequencing technologies are often accompanied by developments of the bioinformatics software to handle these data. It is important then to consider that multiple versions exist for the several software available and the impact it may have on the obtained results. For example, different versions of mapping and alignment processing software can produce different results when performing SNP calling, this depends among others on the dataset used (https://snp-pipeline.readthedocs.io/en/latest/reproducible.html (accessed on 10 December 2020)). From one software version to another, algorithm, parameters used and associated databases might change and then impact the result generated. For example, different allele databases can lead to different results when performing cgMLST analysis [64,65]. Additionally, handler misuse by lack of understanding of those possible changes might also lead to result differences. It is thus important to document and report the versions used to allow other users to reproduce the results and to be aware of the impact that a different version can have on their analyses. Likewise, if the software in the workflow uses a database, it is important to keep these databases updated when possible and to keep a log of the changes/updates.

### 5.6. Needed Expertise and Knowledge

The question that arises prior implementing WGS for pathogen contamination root cause investigation in food factories is: Which kind of expertise is required to use WGS and its results? As the end-to-end workflow encompasses sample taking, sample diagnostics, pathogen isolation, DNA extraction from the isolate, sequencing, bioinformatics analysis and results interpretation, the required know-how should cover these domains. The sequencing laboratory part (DNA extraction, library preparation and sequencing) is frequently outsourced, this does not imply blindly trusting the received data, on the contrary, there should be an additional check to ensure its quality as the specific lab practices are less well known. If ultimately a workflow gets integrated into a pipeline that can be run on a cloud-based server, scripting and programming would not be a must, but understanding the bioinformatics behind (sequence alignment, variant calling, k-mer based typing, etc.) remains an important part to ensure high quality results and to, eventually, perform troubleshooting when needed. As mentioned several times, data is not stand-alone, expertise in microbiology and food safety is necessary to link variant call data and metadata for a comprehensive and reliable interpretation in the factory context. With certain deployments in terms of wet-lab and bioinformatics workflow, it may not be relevant to have a person dedicated to each one of the steps (sequencing, data processing and results interpretation) but overall knowledge of each stage is indeed needed.

In many cases, cloud-based web interfaces (e.g., GalaxyTrakr available at https://www.galaxytrakr.org (accessed on 9 December 2020)) or windows-based commercial software have been developed and deployed. This represents a significative advantage as it enables data analysis without command-line or Linux-based infrastructure, permitting thus a wider adoption, when computing infrastructure or programming expertise is not readily available. It is, however, important to consider that such systems are not “fool-proof”. A critical eye is still needed so the tool does not turn into a black box where the input and output are known but the procedure is not controlled. Often, all stages of the analysis use specific software, each one with multiple parameters and the deployed interfaces offer a higher or lesser control over those parameters that needs to be considered. Neglecting control over these parameters can interfere when comparing runs, even when using the same software and versions. Adding all those quality verifications at each stage should increase the level of confidence in the obtained results.

## 6. Conclusions

The intention is not to consider the described workflow as the new “gold standard” in WGS analyses for pathogen source tracking, but rather present our experiences as food industry and open the discussion about practices to be implemented when using this kind of data. We believe that sharing this stepwise approach with the community is a stage towards standardization of the analysis for robust bioinformatics data analysis and reliable results interpretation. Such a workflow can be implemented in commercial software solutions or in open-source pipelines and would simplify the data analysis for users. Optimization of the workflow allows also for reducing “time-to-result” and indirectly costs. Once agreed upon guidelines will be further defined, more food companies may want to start using WGS in their surveillance programs. Ultimately, this work aims at promoting and facilitating the use of this, highly beneficial technology, to become a routine analysis among the food safety tools already available.

## Figures and Tables

**Table 1:** List of main stages, benchmarked software and parameters considered to ensure high-quality data.

Stage	Evaluated Software	Parameters Considered to Ensure High-Quality Data	Examples of QC Evaluation
Raw reads quality control (QC)	FASTQC [21]	- Per base sequence quality - GC content - Average genome coverage	GC content deviating from expected indicates a possible contamination or sample mislabeling.
Isolate identification	SalmID [22] Sixess [23] KmerID [24] Kraken [25]	- Predicted genus and species of the isolate - Percentage of reads attributed to correct species - Percentage of unclassified reads	A relatively high number of unclassified reads has been associated with plasmid/phage presence, or with a contamination.
Read quality trim and removal	Trimmomatic [26]	- Number of discarded reads - Read length distribution after trimming	A large number of discarded reads was related to poor quality of the sequencing run.
Genome assembly	SPAdes [27] Skesa [28]	Assessed with QUAST [29] - Number of contigs - Genome size - N50	High number of contigs, or deviation of the expected genome size is an indication of low sequenced genome quality [30].
Sequence typing	mlst [31] MLST-CGE [32] stringMLST [33]	- 7 genes MLST composition	Lack of predicted MLST points to low assembly quality.
*Salmonella* serovar prediction *Bacillus* clade prediction	Sistr [34] SeqSero2 [35] BTyper [36]	Serovar/clade prediction [37]	Lack of predicted serovar points to low assembly quality.
First grouping (cg/wgMLST)	chewBBACA [38]	Number of uncalled loci in the genome	A high number of loci from the profile not found in the genome indicates low assembly quality, contamination, or misidentification of the species.
SNP calling	CFSAN SNP pipeline [16]	- Percentage of reads mapped to the reference - Number of SNP missing positions - Differences between raw and preserved matrices.	A large number of missing positions suggest an inappropriate choice of the reference.
Mobile Genetic Elements (MGE) identification such as phages or plasmids	Phigaro [39] ProphET [40] MOB-Suite [41]	- MGE type - MGE position in the genome	Eventually, SNP analyses are run after MGE removal/masking to confirm relatedness.

**Table 2:** List of currently implemented software in our workflow.

Stage	Software
Data integrity	md5sum
Raw reads quality control (QC)	FastQC
Isolate identification	Kraken
Read quality trim and removal	Trimmomatic
Genome assembly	SKESA
Sequence typing	mlst
*Bacillus* clade prediction	btyper
*Salmonella* serovar prediction	SeqSero2
First grouping (cg/wgMLST)	chewBBACA
SNP calling	cfsan_snp_pipeline
Mobile Genetic Elements (MGE) identification such as phages or plasmids	MOB-Suite
	Phigaro
	ProphET
